# Retrospective observation of mental disorders during postpartum period: Results from the Singapore mental health study

**DOI:** 10.1186/s12905-015-0279-x

**Published:** 2015-12-16

**Authors:** Vathsala Sagayadevan, Siau Pheng Lee, Edimansyah Abdin, Janhavi Vaingankar, Helen Chen, Siow Ann Chong, Mythily Subramaniam

**Affiliations:** Research Division, Institute of Mental Health, 10 Buangkok View, Singapore, 539747 Singapore; Department of Psychological Medicine, KK Women’s and Children’s Hospital, 100 Bukit Timah Road, Singapore, 229899 Singapore; Duke-National University of Singapore, 8 College Road, Singapore, 169857 Singapore

**Keywords:** Pre-natal, Post-natal, Women, Mood disorder, Anxiety disorder

## Abstract

**Background:**

The perinatal period has been identified as a period of vulnerability for various disorders (particularly anxiety and depressive disorders), which have been associated with negative outcomes for both mother and infant. The current study utilized data from the Singapore Mental Health Study (SMHS) to examine the temporal relationship between mental disorders and the perinatal period, as well as associated risk factors.

**Methods:**

Life table estimation method was used to derive the estimated hazard rate for any mood or anxiety disorders following pregnancy. Multivariate logistic regression was used to examine the association between socio-demographic factors and onset of mental disorders after the first pregnancy.

**Results:**

Among women with children (*n* = 2278), 1.5 % were found to have an onset of any mental disorder (i.e., mood disorders, anxiety disorders, alcohol use disorders), within 2 years after pregnancy. A peak in hazard rate was noted at approximately 1 year following pregnancy for anxiety disorders but not mood disorders. Women who were married, employed and physically healthy were less likely to have had developed any mental disorder.

**Conclusions:**

The prevalence of mental disorders during pregnancy and postpartum was found to be low among women with children in our community sample, with increased vulnerability following delivery. The results offer some insight into the occurrence of mental disorders during the perinatal period among women in Singapore.

## Background

Physiological, hormonal, psychological, and social role changes make pregnancy a time of potential vulnerability for various disorders [1–4]. A systematic review by Fisher et al. (2012) for instance, found the prevalence of pre- and post-natal disorders in low income countries to be 15.6 and 19.8 % respectively [4]; whereas, Gavin et al. (2005) reported the estimated prevalence of perinatal depression to range between 6.5 and 12.9 %, with 19.2 % of women found to have a depressive episode within the first 3 months postpartum [5].

Several risk factors have been suggested for psychiatric conditions during pregnancy and the postpartum period. These include - but are not limited to - lower socio-economic status, lower educational level, younger age, not being married, lack of support and empathy from partner, domestic violence, and previous psychiatric history [2, 4, 6–12]. Evidence however, has been inconclusive owing to the lack of standardization in survey instruments and the use of different cutoff points for screening tools in determining clinically significant symptomatology [8].

Anxiety and depressive disorders in particular, have received extensive attention in the area of pre- and post-natal research given their high co-morbidity [13] and the adverse effects that these disorders have on both the mother and the infant [1, 4, 6]. With regards to postpartum depression, antenatal depression or anxiety [2, 6, 8] has been consistently implicated as a significant risk factor. In a multicenter prospective study among Japanese women for instance, Kitamura et al. [14] found antenatal depression to be a significant risk factor for postnatal depression. This finding was replicated by Leung et al. [15] among postpartum women in Hong Kong whereby antenatal depression (and perceived stress) significantly predicted an approximate 28 % of the variance in postpartum depression. Research has alluded this to women with antenatal depression being more prone to experiencing a difficult labor and distress about the birth which in turn increases the likelihood of postpartum depression [16].

Antenatal anxiety on the other hand, has been linked to physical defects in the infant, low birth weight, and poor cognitive and neurodevelopment. Anxiety disorders/symptoms are commonly reported during the pre-natal period during which the mother may be concerned about the impending delivery of the child [17] and the associated changes in social roles and responsibilities that this brings forth [7]. In particular, obsessional thoughts pertaining to fear and concern of causing harm to the infant are common [18]. This phenomenon has been postulated to be especially pronounced in an Asian context given the various taboos associated with childbirth and the expectation of Asian women to follow certain traditions to ensure the well-being of the infant [19].

Although pre- and post-natal research has been conducted in the local population, studies are relatively limited. Thiagayson et al. [20] for instance, found the rates of major depression, minor depression, and anxiety disorder to be 11, 7, and 12.5 % respectively among a sample of Singaporean women who were at high obstetric risk. Conversely, in a prospective cohort study by Chee et al. [21], almost one-third of Singaporean women who engaged in the postpartum convalescence period (also referred to as “doing the month” for 30 days after childbirth) quoted it as negative experience which in turn significantly contributed to their depressive episode.

Given the limited research with regards to pre- and post natal psychiatric disorders particularly within the local context, the current paper aims to further build on the understanding of the epidemiology of women’s reproductive mental health by utilizing data from the Singapore Mental Health Study (SMHS) to explore the temporal relationship of pregnancy and child birth on mental disorders (i.e., mood disorders, anxiety disorders, alcohol use disorders). Furthermore, given the mixed evidence with regards to socio-demographic factors as potential risk factors for postnatal mental disorders, the study aims to examine this in the multi-ethnic population of Singapore as this may aid clinicians in identifying individuals who may be at a higher risk of developing psychiatric conditions during this period.

## Methods

### Study design and sampling

Singapore is a multi-ethnic country in Southeast Asia with a population of 5 million. 74.2 % of the population is of Chinese ethnicity, 13.3 % are Malay, 9.1 % are Indian, and 3.3 % belong to other ethnic groups [22]. Singapore ranks relatively high in terms of Human Development Index (defined as ‘average achievements in a country on the basis of three dimensions of human development: life expectancy, years of schooling, Gross national income (GNI) per capita) and ranks low in terms of Gender-related Development Index (GDI), an index designed to measure gender inequality across the aforementioned three dimensions [23]. Singapore also has a relatively well-developed health care system, whereby most women receive obstetric-led care and deliver their babies in hospitals [24, 25].

The SMHS was a nationwide epidemiological survey conducted in Singapore between December 2009 and December 2010. A total of 6616 respondents, aged 18 years and older were recruited for the survey. Disproportionate stratified sampling was used to sample an equivalent proportion of 30 % of the three main ethnic groups in Singapore (Chinese, Malays, and Indians). Respondents were randomly selected from a national registry, and were approached at their households for face-to-face interviews. The study was approved by the relevant institutional ethics committee (National Healthcare Group, Domain Specific Review Board). A response rate of 75.9 % was achieved in the study. All participants and parents of participants aged 18–20 years (age of majority in Singapore is 21 years) provided written informed consent prior to study participation. The detailed methodology of the current survey has been described in an earlier article [26].

### Instruments

#### Socio-demographic information and child-related variables

Socio-demographic information was collected using a structured questionnaire. This included age of woman at the time of interview, ethnicity (Chinese, Malay, Indian, and other), income level (< 20,000 SGD, 20,000–49,999 SGD and ≥ 50,000 SGD), marital status (never married, married, divorced or separated and widowed), educational level and employment status (employed, economically inactive which included students and housewives and unemployed). Details of the number of biological children and the age of the first biological child were also collected.

#### Composite international diagnostic interview version 3.0

##### Diagnoses of mental disorders

Diagnoses of mental disorders were established using the World Mental Health Composite International Diagnostic Interview version 3.0(CIDI-3.0) [27]. The CIDI 3.0 assesses both lifetime and 12-month prevalence of disorders using the definitions and criteria of the DSM-IV and the International Classification of Disease (ICD-10). Diagnostic modules for lifetime prevalence of mood disorders, including major depressive disorder (MDD), dysthymia and bipolar disorder; anxiety disorders, including generalized anxiety disorder (GAD), and obsessive compulsive disorder (OCD); and alcohol use disorders were included in the survey. The CIDI has been administered to respondents from various cultural and educational backgrounds [28] and has established reliability and validity [29, 30].

##### Chronic medical conditions

A modified version of the CIDI checklist of chronic medical conditions was used to assess the presence of the following conditions: cancer, cardiovascular disease, chronic pain, diabetes, high blood pressure, neurological conditions, and ulcer.

### Data analysis

Of the 3317 female respondents in the SMHS, 2278 reported having biological children. The age at first pregnancy was deduced by first subtracting the age of the first biological child from the woman’s age and further deducting 1 year from this to account for the duration of pregnancy. Statistical analyses were carried out using the Statistical Analysis Software (SAS) System version 9.2. All estimates were weighted to adjust for over-sampling and post-stratified for age and ethnicity distributions between the survey sample and the Singapore resident population in 2007.

Life table estimation (using proc lifetest method) was used to derive the estimated hazard rate for any mood or anxiety disorders, with the time since pregnancy as the starting time point. Multivariate logistic regression was used to examine the association between socio-demographic factors and the onset of mental disorders after pregnancy. Socio-demographic factors including age group, ethnicity, marital status, education level, presence of chronic physical conditions, employment status and income level were entered in the same block for the logistic regression.

## Results

### Prevalence

In the current sample, the mean probable age at first pregnancy was 25.6 (95 % CI: 25.3–25.9) years. Socio-demographic characteristics of the study sample are reflected in Table 1. Of the females in the general population (50.1 %, *n* = 3317), 11.38 % (*n* = 406, S.E. = 0.8) were found to have an onset of any mental disorder; 8.38 % (*n* = 291, S.E. = 0.7) reported mood disorders, 3.98 % (*n* = 152, S.E. = 0.5) had anxiety disorders, and 1.44 % had alcohol use disorders (*n* = 50, S.E. = 0.27). Of these respondents, 66.8 % (*n* = 2278), had children; among whom, 9.43 % (*n* = 228, S.E. = 0.9) had an onset of any mental disorder; 7.15 % (*n* = 163, S.E. = 0.8) reported mood disorders, 3.61 % (*n* = 92, S.E. = 0.5) had anxiety disorders, and 0.50 % had alcohol use disorders (*n* = 16, S.E. = 0.2).

**Table 1:** Socio-demographic characteristics of the study sample

		Onset of mental disorder after pregnancy		Onset of mental disorder within 2 years after pregnancy	
		*n*	%	*n*	%
Age group	18–34	41	9.02	22	5.87
	35–49	70	6.76	12	1.74
	50–64	35	5.54	0	.
	65+	3	4.26	0	.
Ethnicity	Chinese	42	6.31	10	1.43
	Malay	48	5.20	10	1.13
	Indian	54	8.46	13	2.30
	Others	5	9.39	1	2.35
Marital Status	Married	115	4.75	27	1.17
	Never married / single	2	18.55	1	6.15
	Divorced/ Separated	25	24.09	6	6.97
	Widowed	7	7.77	0	.
Education	University	18	5.69	9	3.81
	Pre-U /Junior College / Diploma	34	10.74	9	2.74
	Vocational	11	14.79	7	9.95
	Secondary	60	7.34	6	0.52
	Primary	23	2.42	3	0.20
	Pre-Primary	3	3.27	0	.
Any Chronic Physical Condition	No	69	5.65	16	1.41
	Yes	80	7.27	18	1.53
Employment Status	Employed	85	6.68	23	2.17
	Economically Inactive	49	5.43	9	0.59
	Unemployed	10	14.24	1	0.45
Income	Below S$20,000	89	5.53	17	0.59
	S$20,000–S$49,000	40	8.55	9	2.21
	Above S$50,000	10	8.35	6	5.56

Among female respondents with children (66.8 %, *n* = 2278), 1.5 % (*n* = 34, S.E. = 0.4) were found to have an onset of any mental disorder, within 2 years of the first pregnancy; 0.9 % (*n* = 19, S.E. = 0.3) reported mood disorders, 0.7 % (*n* = 14, S.E. = 0.3) had anxiety disorders, and 0.02 % had alcohol use disorders (*n* = 2, S.E. = 0.02). Among individuals with mood disorders, 0.5 % (*n* = 11, S.E. = 0.2) had major depressive disorder (MDD), 0.1 % (*n* = 3, S.E. = 0.1) had dysthymia, and 0.3 % (*n* = 6, S.E. = 0.2) had bipolar disorder. Among those with anxiety disorders, 0.2 % (*n* = 2, S.E. = 0.2) had generalized anxiety disorder (GAD), and 0.5 % (*n* = 12, S.E. = 0.2) had obsessive compulsive disorder (OCD). The 2 year period is critical as it incorporates pregnancy, giving birth, and the period involving infant care.

Of those who had a child, 6.4 % (*n* = 149, S.E. = 9.8) were observed to have an onset of a mental disorder at any time point probable after the first pregnancy. Of these, 5.2 % (*n* = 113, S.E. = 0.7) had mood disorders, 1.8 % (*n* = 46, S.E. = 0.4) had anxiety disorders, and 0.1 % (*n* = 6, S.E. = 0.05) had alcohol use disorders (Table 2).

**Table 2:** Prevalence of DSM IV mental disorders among females who have at least one child

	Onset at any time point after first pregnancy			Onset within 2 years after first pregnancy		
	*N*	%	SE	*N*	%	SE
Major Depressive Disorder	91	4.0	0.6	11	0.5	0.2
Dysthymia	12	0.4	0.2	3	0.1	0.1
Bipolar Disorder	21	1.1	0.3	6	0.3	0.2
Generalized Anxiety Disorder	14	0.6	0.2	2	0.2	0.2
Obsessive Compulsive Disorder	34	1.2	0.3	12	0.5	0.2
Alcohol Abuse	2	0.0	0.0	1	0.0	0.0
Alcohol Dependent	4	0.1	0.0	1	0.0	0.0
*Mood Disorders*	113	5.2	0.7	19	0.9	0.3
*Anxiety Disorders*	46	1.8	0.4	14	0.7	0.3
*Addictive Disorders*	6	0.1	0.1	2	0.0	0.0
*Any disorder*	149	6.4	0.8	34	1.5	0.4

### Estimated hazard rate

**Fig. 1:**
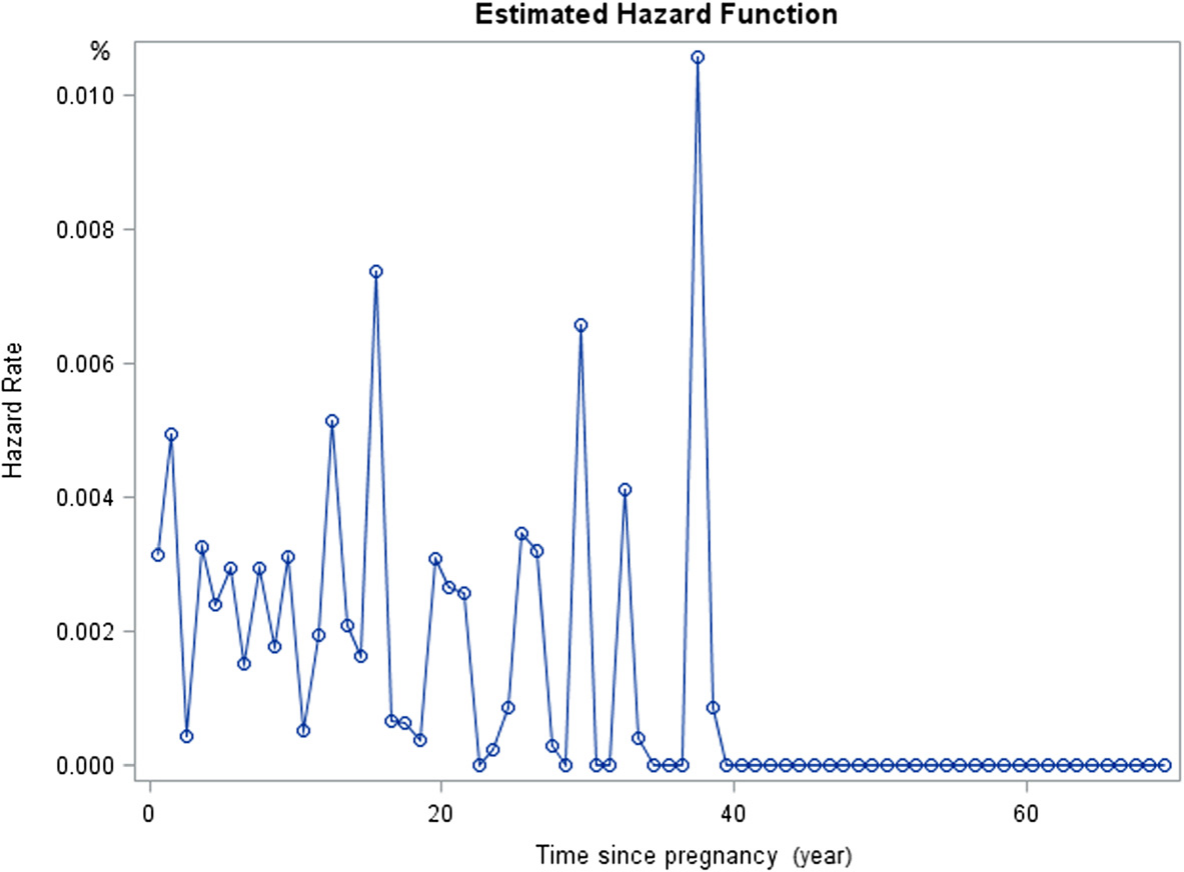
Estimated Hazard of Any Mood Disorders since Pregnancy

**Fig. 2:**
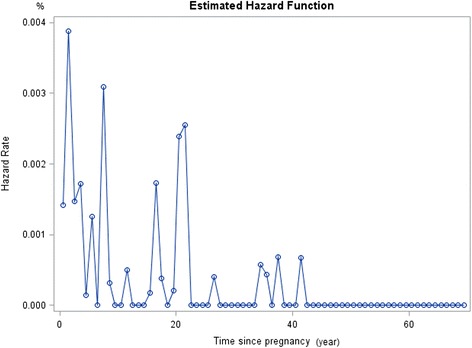
Estimated Hazard of Any Anxiety Disorders since Pregnancy

With reference to the life-table of age of onset of mental disorders, the estimated hazard rate of occurrence of mood disorders following pregnancy was relatively constant (Fig. 1). A peak in hazard rate was however noted at approximately 1 year following pregnancy for anxiety disorders (Fig. 2).

### Socio-demographic correlates and onset of mental disorders

Compared to married women, divorced and separated women had higher odds (OR = 22.1; 95 % CI; 5.7, 86.5) of onset of any mental disorder within the 2 years of the first pregnancy. Likewise, the presence of any chronic physical condition was found to be associated with higher odds of onset (OR = 3.1; 95 % CI; 1.1, 8.9).

Marital status stood out as a risk factor for the occurrence of a mental disorder at any time point after a woman’s first pregnancy. Divorced or separated (OR = 9.2; 95 % CI; 4.6, 18.5), and widowed women (OR = 3.5; 95 % CI; 1.4, 8.6) had higher odds of having an onset of mental disorder after pregnancy compared to married women. In particular, divorced or separated (OR = 10.1; 95 % CI; 4.8, 21.3), and widowed women (OR = 3.9; 95 % CI; 1.5, 9.7) were found to have higher odds of onset of mood disorders, following pregnancy. Additionally, being unemployed (OR = 4.7; 95 % CI; 1.1, 20.9) as opposed to being employed was found to be associated with higher odds of having an onset of mental disorder at any time point after pregnancy.

## Discussion

The findings from this study suggest a considerably lower prevalence of mental disorders during pregnancy and postpartum period among women who have children compared to that reported in other studies (e.g. [4, 5]). These rates were low even when compared to estimates reported in Asian countries. Klanin et al. [12] for instance, reported prevalence of postpartum depression to range between 3.5 and 63.3 % in Asian countries.

One reason for the low prevalence of postpartum depression among Singaporean women as compared to other Asian countries could be due to Singapore ranking relatively higher in terms of HDI compared to other countries [23]. The high level of development not only allows individuals to have access to good quality healthcare services but also allows them to seek proper treatment for these psychiatric conditions. This is in contrast to low-income and low-middle income countries whereby poverty not only limits access to proper and timely health care but also restricts their ability to afford these services [31]. Likewise, greater gender equality in terms of access to education and command over economic resources also means that females in Singapore are in a more privileged position to be able to afford and utilize these healthcare services relative to lower income countries whereby gender disparity may limit the autonomy of women in seeking out these services [31].

In contrast to our study, Chee et al. [21] found 18 % of pregnant women in Singapore to have either major or minor depression and 12.5 % to have anxiety disorders, which were relatively higher than our finding of 0.9 % for mood disorders and 0.7 % for anxiety disorders. One possible reason for this discrepant finding between Chee et al. [21] and our finding is likely due to the use of different instruments in assessing mental disorders. While our study utilized the CIDI to establish diagnoses of mental disorders including depression, Chee et al. [21] used the Edinburgh Postnatal Depression Scale (EPDS) designed specifically to measure postpartum depressive symptoms. Though well-validated, the CIDI is not specifically developed to assess psychiatric symptoms associated with disorders during pregnancy and postpartum [32]. For instance, particular symptoms such as changes in appetite or weight, fatigue, sleep disruptions in the CIDI have been reported to be poor in terms of discriminating between clinical depression and ‘normative experience of pregnancy’ given the overlap of symptoms between these two [11, 32]. Thus, while severe cases of postnatal depressions could have been detected, less severe presentations could have been overlooked as natural consequences of childbirth [33]. Furthermore, given some evidence to suggest the use of clinical interviews to yield lower prevalence rates compared to self-report measures, the rates in the current study could have been underestimated to some extent [34].

Conversely, past studies have suggested some difference in symptom expression between the Western and Asian populations. For example, Oates et al. [35] found Asians to have a higher tendency to somatize their symptoms compared to the Western population whereas Hwu et al. [36] postulated Asian cultures to have a ‘relatively high tolerance for or denial of emotional sufferings’ (also known as cultural stoicism). In line with this, Liao et al. [37] reported the prevalence of MDD symptoms to be lowest among non-institutionalized Taiwanese adults followed by Koreans and Americans (ECA and NCS study) [38]. Given that the CIDI has been primarily validated for use in the Western populations [39], the tendency for Asian individuals to repress their emotions as well as somatize symptoms may have resulted in a response bias [37] which may in turn have resulted in lower estimates of mental disorders obtained in this study.

Likewise, the lower prevalence could also have been a result of under-reporting due to a lack of awareness of mental illness, reluctance to report symptoms to a stranger due to shame or stigma [16, 39] as well as recall bias, whereby the women may have been overwhelmed by the tasks of taking care of the infant, and other changes in their life that they have “sealed over” any occurrence of mental health issues.

Despite the low prevalence of mental disorders during pregnancy and the post-partum period, the current findings were in line with past literature which has shown the prevalence of mood and anxiety disorders to be relatively higher during the postpartum period [5, 27]. For instance, though the prevalence of onset of any mental disorder was higher among women in the general population compared to women with children, the prevalence of both mood and anxiety disorders were comparable in both populations.

The onset of anxiety disorders in the current study was found to be associated with the time of pregnancy and particularly in the postpartum, with OCD being the most prevalent mental disorder within 2 years after a woman’s pregnancy. This finding corroborated with past research which have shown anxiety disorders to be common during the postpartum period [18, 27, 40, 41]. Evolutionary theories for instance, posit that intrusive thoughts and obsessional thoughts may to some extent represent adaptive behaviors meant to protect their infants from possible harm [40, 42]. The current finding of high prevalence of OCD was also partially supportive of an earlier study conducted by Chong et al. [39] who found the prevalence rates of mental disorders, with the exception of OCD in the adult Singapore resident population to be considerably lower than those in western countries. Similarly, Subramaniam et al. [43] also found the prevalence of obsessions and compulsions to be higher in the Singapore adult population compared to the US. The relatively higher prevalence in OCD observed in both the general and post-partum female population might suggest a higher risk for this disorder among the local population and warrants further investigation.

With regards to socio-demographic variables, only marital status and employment stood out as significant factors in influencing women’s mental health in the current study. Divorced and separated women were more likely to have an onset of mental disorder after pregnancy, or within the 2 year period following pregnancy, compared to married women. These findings paralleled those of Subramaniam et al. [44], who found single mothers to have significantly higher odds of having mood disorders as compared to those who were married. Previous studies have found the role of social support, particularly support provided by spouses to be a protective factor against mental disorders during this critical period [4, 45]. This finding should however be interpreted with caution, as marital status was obtained at the time when the respondent was being surveyed and as such may not accurately reflect the marital status of respondents at the time of pregnancy and during the postpartum period. Furthermore, it is important to note that the finding implies a correlational rather than a cause-effect relationship and that the onset of mental disorder may predate the change in marital status.

Absence of chronic physical conditions and being employed also served as protective factors for women in the current sample. Women who were married, employed and physically healthy were less likely to have developed any mental disorder than those who were not. This was in line with past studies which have shown low socio-economic status and financial difficulties to be strongly associated with postnatal psychiatric disorders [46, 47].

In contrast to past local studies, ethnicity was not found to be significantly associated with the onset of mental disorders. Chong et al. [48] for instance, in examining the prevalence of depressive symptoms among outpatients with diabetes in Singapore, found Indian ethnicity to be significantly associated with a high risk of depression. Similarly, Chong et al. [39] found the prevalence of depression to be low among adult residents, particularly the Chinese as opposed to other ethnic groups. We are however unable to account for the negative finding in this study.

## Limitations

There are limitations in our study. We were unable to examine the effect of miscarriage, abortion, child death, and gender-based violence on females’ mental health in the current study. Past literature for instance, has suggested a link between gender-based violence (including emotional and physical abuse) during pregnancy and the incidence of psychiatric disorders [4, 49]. Relatively prevalent among socially and economically disadvantaged women, factors such as bias against female babies, role restrictions, as well as multi-generational households in which the daughter-in-law has little autonomy have been identified as possible risk factors for gender-based violence [4]; these factors however were not explored in this study. We were also unable to detect the cases of subclinical levels of depression, and panic disorder as the study did not establish these diagnoses. Estimation of age at first pregnancy was an approximate made from subtracting the age of the first biological child from the respondent’s age and further deducting 1 year (as opposed to 9–10 months to account for pregnancy). Given that not all pregnancies last 9 months, this may have resulted in an over-estimation of the prevalence of mental disorders during the postnatal period. Furthermore, as the CIDI does not contain a specific question targeted at female respondents with regards to reproductive events, we were not able to gauge a more precise pregnancy age. Additionally, we were not able to differentiate the effect of ageing and child birth on respondents’ mental health status, all of which are important factors that can impact women’s mental health [50]. The retrospective design of the current study also limits inference of causality. The low prevalence in the current sample warrants careful interpretation of results given the insufficient statistical power. Future studies can look into utilizing a prospective study design to better understand the impact of pregnancy on women’s mental health.

## Conclusions

The prevalence of mental disorders during the two-year period following pregnancy was found to be low in the current sample. However, a peak in estimated hazard rate for anxiety disorders was noted at the time of giving birth. Being married, employed, and the absence of chronic physical conditions (i.e., better physical health) were found to be protective factors for the mental health of women who have children. While the results offer some insight into the impact of pregnancy and child birth on women’s mental health in an Asian population, further studies based on a prospective or follow-up design may help to better understand these associations.

## Abbreviations

SMHS: Singapore Mental Health Study
CIDI-3.0: Composite International Diagnostic Interview version 3.0
ICD-10: International Classification of Disease
MDD: Major depressive disorder
GAD: Generalized anxiety disorder
